# Dasatinib-Induced Bilateral Pleural Effusions

**DOI:** 10.7759/cureus.23906

**Published:** 2022-04-07

**Authors:** Taha F Rasul, Gabriel Motoa, Robert C Flowers

**Affiliations:** 1 Internal Medicine, University of Miami Miller School of Medicine, Miami, USA; 2 Internal Medicine, University of Miami Miller School of Medicine, Jackson Memorial Hospital, Miami, USA

**Keywords:** treatment, chronic myeloid leukemia, pleural effusions, bilateral, dasatinib

## Abstract

Fluid accumulation in the form of pleural effusions and ascites may be attributed to a single etiology. Diagnosis depends on a thorough clinical history as well as fluid analysis. We present the case of a 60-year-old man with chronic myeloid leukemia (CML) on dasatinib, recent right-sided ischemic stroke, alcohol-associated liver disease, cocaine and alcohol use disorders in early remission, and hypertension who presented with subacute-onset of bilateral pleural effusions and ascites. Pleural fluid analysis showed an exudative effusion, while ascitic fluid analysis showed a transudative collection. After an extensive workup, the bilateral effusions were attributed to dasatinib therapy, which was also suspected to play an unclear role in the worsening ascites. Although peripheral edema and pleural effusions are well-recognized and common side effects of tyrosine kinase inhibitors (TKIs), this case represents the first description of a patient presenting with bilateral TKI-induced pleural effusions as well as concomitant ascites of unclear origin.

## Introduction

Dasatinib is a tyrosine kinase inhibitor (TKI) used in the treatment of imatinib-resistant breakpoint cluster region-Abelson murine leukemia (BCR-ABL)-positive chronic myeloid leukemia (CML). Like other TKIs, it is generally well-tolerated with common adverse effects including peripheral edema and pulmonary edema in 10%-22% and 15%-35% of patients, respectively [1]. Notably, pleural effusions attributed to dasatinib are characterized as exudative effusions and usually occur unilaterally [2]. TKIs can cause pleural effusions through reactive oxygen species causing increased endothelial permeability [3]. In theory, this same process could occur in the splanchnic and hepatic vasculature and lead to ascites. Although the exact mechanism is not well-understood, it may also involve inhibition of platelet-derived growth factor receptor beta (PDGFRB) or an immune-mediated reaction. Currently, it is not known which mechanism has the largest role in TKI-induced fluid retention [3].

New-onset pleural effusions or ascites require direct sampling to determine the cause. For pleural effusions, evaluations of protein and lactate dehydrogenase (LDH) are used. Light’s Criteria utilizes the ratio of protein and LDH in the pleural fluid compared to that in the serum [4]. Exudative effusions are generally characterized by pleural-to-serum protein ratios of greater than 0.5, and pleural-to-LDH ratios over 0.6 (or two-thirds of the upper limit of normal serum LDH). Such effusions may be seen in infections, malignancies, and pulmonary emboli. Transudative effusions have characteristic protein ratios below 0.5 and LDH ratios below 0.6 (or less than two-thirds of the upper limit of normal serum LDH) and are seen in conditions like congestive heart failure, hepatic cirrhosis, and nephrotic syndrome. Ascites are evaluated with the serum ascites albumin gradient (SAAG) and by comparison of the ascitic and serum total protein levels [5]. High SAAG, or transudative ascites, indicates extrusion of mostly fluid from the vasculature into the peritoneal cavity and can occur from conditions such as cirrhosis and right heart failure. Low SAAG, or exudative ascites results from a decreased intravascular osmotic gradient leading to a downstream influx of proteinaceous fluid from the intravascular to peritoneal space. This is seen in conditions like malignancy, pancreatitis, and hypoalbuminemia. In addition to the above etiologies of pleural effusions and ascites, there are other well-described drug-induced mechanisms for these presentations. These include sodium overload (such as corticosteroids), renal dysfunction (non-steroidal anti-inflammatory drugs), and hyperpermeability of blood vessels (vasoactive agents including vasopressors) [6].

Ascitic fluid may cause predominantly right-sided pleural effusions due to proximity to the diaphragm as well as intradiaphragmatic rents [7]. This has been described in conditions such as disseminated tuberculosis, advanced malignancy, and pancreatitis [8]. We present a case of new-onset bilateral pleural effusions attributed to dasatinib, with potential exacerbation of ascites.

## Case presentation

A 60-year-old man presented to the emergency department (ED) with new-onset abdominal distension and progressive shortness of breath. He had a past medical history of CML, alcohol-associated liver disease, acute ischemic stroke three months prior with residual left hemiparesis, cocaine and alcohol use disorder in early remission, obstructive sleep apnea, gout, hypertension, anxiety, and depression. He had noticed gradually increasing abdominal girth and weight gain over the preceding three weeks despite complete alcohol cessation following his ischemic stroke. He also reported a loss of appetite, orthopnea, and weight gain but denied melena, hematochezia, fever, chills, cough, or hemoptysis. His home medications included dasatinib (which he had been taking for 3-4 months), metoprolol, amlodipine, allopurinol, calcium carbonate, folic acid, and thiamine. Upon arrival to the ED, he was afebrile, mildly hypertensive to 150/78 mm Hg, with a heart rate of 85, respiratory rate of 18, and peripheral oxygen saturation of 91% on room air. Physical exam was notable for bibasilar decreased breath sounds and crackles at the mid lung fields. His abdomen was distended and non-tender with shifting dullness. No pedal edema was noted. There was the chronic motor weakness of the left upper extremity. No signs of liver cell failure were noted.

**Figure 1:**
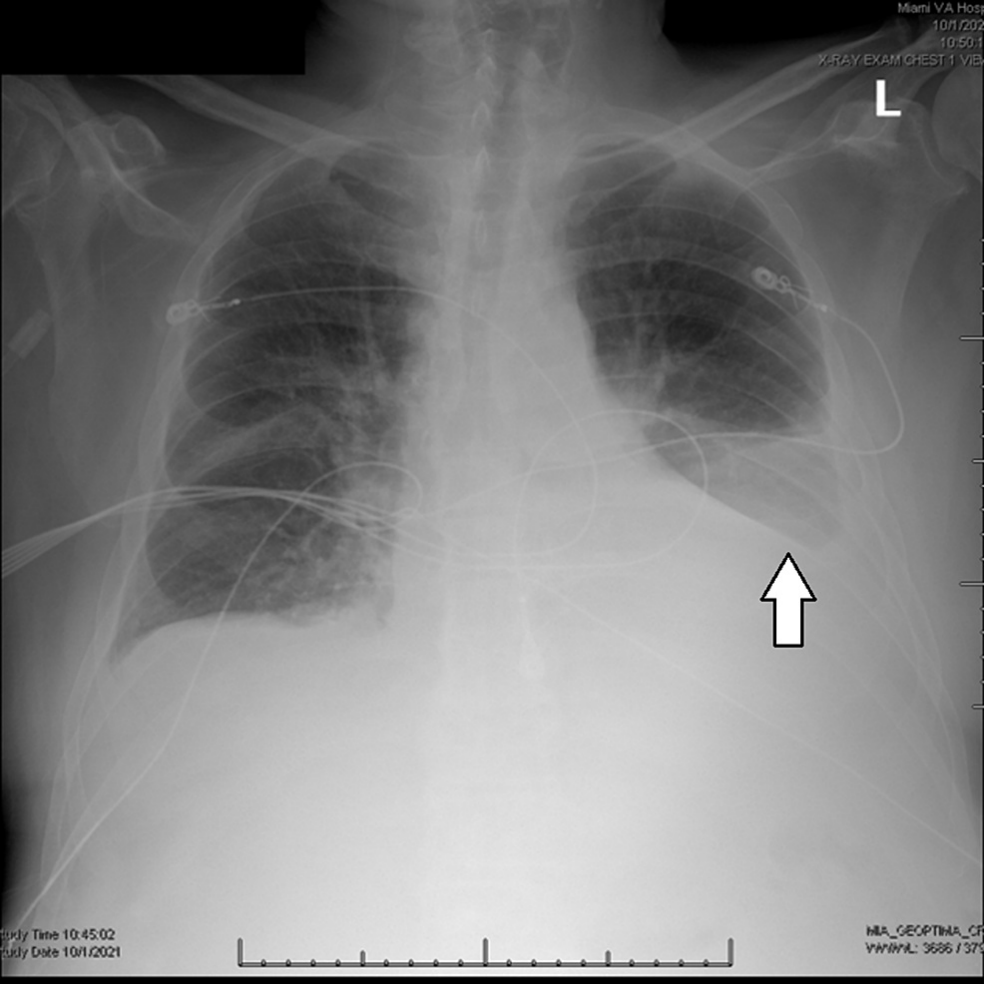
Posteroanterior chest radiograph on admission showing congestive changes, left-sided pleural effusion (arrow), and basilar underaeration.

Serum laboratory analysis was notable for hemoglobin 12.3 mg/dL and potassium 3.6 mEq/dL. Liver function panel and aminotransferase levels were within normal limits. Workup of liver disease showed normal ceruloplasmin, antinuclear antibody, alpha-1 antitrypsin, hepatitis A and hepatitis B serologies consistent with vaccination, and borderline elevated anti-smooth muscle antibodies at a titer of 1:21, where titers above 1:20 are considered positive. Electrocardiogram and troponin levels were negative for signs of acute cardiac ischemia. Initial chest x-ray (Figure 1) showed congestive changes as well as left-sided pleural effusion and basilar underaeration. 

The patient was admitted for new-onset ascites. He underwent bedside paracentesis which yielded approximately 5 liters of fluid, with a cell count and differential not consistent with spontaneous bacterial peritonitis (Table 1).

**Table 1:** Ascitic fluid analysis

Color	Yellow
Turbidity	Hazy
Protein	3.8 g/dL
Albumin	2.3 g/dL
Albumin (serum)	4.1 g/dL
White blood cell (WBC) count	765 cells/mm^3^
Gram Stain	Negative
Culture	Negative

**Figure 2:**
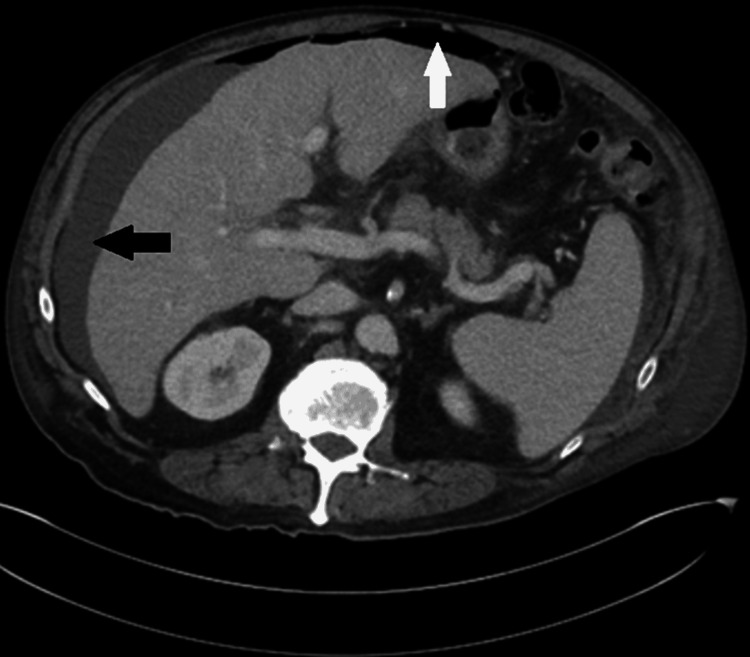
Post-paracentesis abdominal CT showing residual ascites (black arrow) and free air (white arrow), likely post-procedural. The presence of nodular liver contour indicative of cirrhosis, splenomegaly, as well as small gastroesophageal varices.

Post-paracentesis computed tomography (CT) of the abdominal and pelvis (Figure 2) was notable for a nodular liver contour indicative of cirrhosis, splenomegaly, and small gastroesophageal varices.

**Figure 3:**
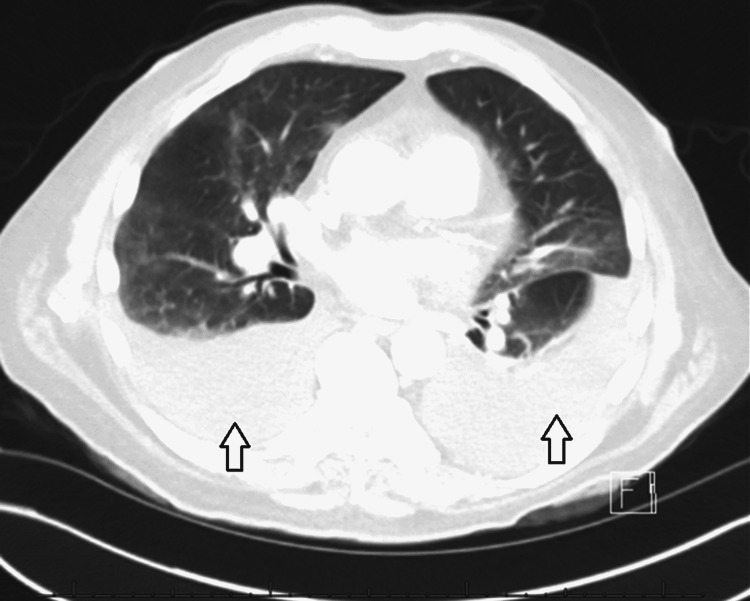
Pre-thoracentesis chest CT showing moderate bilateral opacities (arrows) with mild patchy opacity and bronchiectasis in the right upper lobe, potentially scarring or fibrosis.

Transthoracic echocardiogram showed normal left ventricular ejection fraction, grade 1 diastolic dysfunction, and moderate to severe aortic stenosis. He did not display any clinical signs of aortic stenosis such as syncope and angina. CT of the chest (Figure 3) confirmed large bilateral pleural effusions.

The patient underwent bilateral thoracentesis and pleural fluid samples were sent for analysis (Table 2).

**Table 2:** Pleural fluid analysis

Variables	Patient pleural fluid	Reference values
Color	Amber	Straw
Turbidity	Cloudy	Clear
WBC count	0.0 cells/mm^3^	<1,000/mm^3^
Mesothelial cells	27%	0-3%
Macrophages	13%	59-91%
Protein	3.8 g/dL	1-2 g/dL
LDH	158 U/L	Less than 50% of plasma (Patient LDH was 185 U/L)
Glucose	95 mg/dL	Similar to plasma (Patient glucose was 122 mg/dL)
Amylase	46	30-110 U/L
pH	8.00	7.60-7.64

The patient was treated for symptomatic pleural effusions and ascites while their etiology was determined. He underwent paracentesis with approximately 5L removed and bilateral thoracentesis with approximately 1L removed from each hemithorax. Following drainage, he was able to be weaned from supplemental oxygen. The patient was also managed with diuretic therapy, a low salt diet, and fluid restriction. Diuresis was discontinued upon resolution of the pleural effusions. Concurrent cirrhosis likely contributed to the development of ascites, so treatment with spironolactone was also initiated. Physical therapists regularly visited him to maintain mobility and promote lymphatic drainage. After extensive multidisciplinary discussions with hematology, pulmonology, and hepatology, the patient was diagnosed with dasatinib-induced pleural effusions, and the decision was made to decrease the dasatinib therapy dose.

## Discussion

After thoracentesis, pleural fluid showed elevated protein and LDH suggesting an exudative etiology (Table 2). In the absence of infectious or malignant causes, and given the simultaneous onset of the bilateral pleural effusions and the ascites, it was considered that they were induced by a systematic process. The patient’s SAAG was 1.8, indicating a transudative fluid. The ascitic fluid total protein level was 3.8 g/dL (well above the generally accepted 2.5 g/dL cutoffs), indicative of cardiogenic rather than nephrotic ascites (Table 1). In the absence of portal vein thrombosis and overt cirrhosis, the next differential diagnosis was right heart failure. A transthoracic echocardiogram did not show right or left ventricular systolic dysfunction to support cardiogenic ascites, and the patient did not exhibit other signs or symptoms suggestive of this condition.

An exudative pleural fluid should not occur in the presence of inflammatory conditions which would increase vascular permeability [9]. The absence of infection, lung malignancy or metastatic disease, or autoimmune disorder indicated that the most likely factor was a systematic agent such as dasatinib. Additionally, the established tendency of TKIs to cause exudative pleural effusions further made medication side effects a likely cause. The cause of the ascites, however, was less clear particularly given the patient’s new diagnosis of cirrhosis. Since the patient had a magnetic resonance imaging (MRI) study following his stroke three months prior which showed no evidence of cirrhosis, and experienced complete alcohol cessation since there remained diagnostic uncertainty as to the etiology of the ascites. The CT abdomen findings of gastroesophageal varices and splenomegaly were indicative of portal hypertension (Figure 2). However, our patient had not consumed alcohol for at least three months before the onset of the ascites. Even though alcohol-associated cirrhosis could have been the underlying cause, the near-simultaneous onset of the pleural effusions and ascites, as well as normal transaminases, suggest a possible systemic process. Since ascites secondary to cirrhosis would not be expected to cause exudative pleural effusions, dasatinib’s systemic effects could have extended to the peritoneum. Although increased vascular permeability would be expected, it remains to be seen the exact effects of TKIs on visceral hemodynamics. Therefore, if dasatinib was not the sole inciting factor, it could have had an additive or synergistic effect with cirrhosis on the development of ascites.

Management of TKI-induced pleural effusions is usually symptomatic until stabilization, followed by dose reduction or alternate medications [10]. In cases of refractory effusions, alternate TKIs like nilotinib may be used [11]. For our patient, the post-stabilization management was through a dose reduction of dasatinib rather than switching to another medication. While our patient did receive diuresis with furosemide and spironolactone, it is important to note that the benefit of diuretics to manage dasatinib-induced pleural effusions is unclear [12].

According to recent literature, decreasing the dose by a moderate amount is likely to prevent further edema [13]. It also ensures continuity of CML treatment, particularly in those responding well to dasatinib. For example, in this patient, the bcr-abl/ABL-Ia ratio at the beginning of CML treatment was quantified at 27.9% and decreased to 0.029% after three months of therapy. In patients with a good response to therapy, the most desired approach is decreasing the dose rather than discontinuing the medication altogether. In situations where there is a suboptimal response to the antileukemic agents and dose reduction is not feasible, it may be more beneficial to switch patients to once-daily dosing instead of twice-daily [14,15]. It has been suggested that dasatinib confers an elevated risk for pleural and pericardial effusions, even at dosages of 50 to 100 mg daily [16].

The patient experienced significant symptomatic improvement with the above therapies. He was able to work through physical therapy without dyspnea on exertion. He remained off dasatinib for the duration of his hospitalization of 13 days. The outpatient plan per hematology was to closely monitor the patient after dasatinib dose adjustment, especially due to his high-risk Sokal score. He is currently being treated with dasatinib at a reduced dose, from 100 mg to 70 mg daily. Since discharge, he was seen by hepatology and recommended to continue furosemide as well as further variceal screening. Metoprolol 50 mg was also continued, even though propranolol and nadolol are generally more efficacious at preventing variceal bleeds [17].

## Conclusions

Dasatinib-induced pleural effusions are a relatively common side effect in patients and have been described as exudative by Light’s criteria. Bilateral pleural effusions often represent an induced, generalized state of increased vascular permeability. The exact mechanism of fluid retention due to dasatinib is not well understood and requires further evaluation. Ascites have not been described as a side effect of TKIs, but they may exacerbate ascites in the setting of liver disease. Additionally, TKIs may increase the likelihood of developing ascites due to their potential for fluid retention. Common and life-threatening etiologies of pleural effusions including cardiac, infectious, renal, and malignant causes should be ruled out. Medication-induced pleural effusions are a diagnosis of exclusion. Further prospective studies are necessary to evaluate the possible association of TKIs with new-onset or worsening ascites.

## References

[1] Shah NP, Rousselot P, Schiffer C (2016). Dasatinib in imatinib-resistant or -intolerant chronic-phase, chronic myeloid leukemia patients: 7-year follow-up of study CA180-034. Am J Hematol.

[2] Cortes JE, Jimenez CA, Mauro MJ, Geyer A, Pinilla-Ibarz J, Smith BD (2017). Pleural effusion in dasatinib-treated patients With chronic myeloid leukemia in chronic phase: identification and management. Clin Lymphoma Myeloma Leuk.

[3] Latagliata R, Breccia M, Fava C (2013). Incidence, risk factors and management of pleural effusions during dasatinib treatment in unselected elderly patients with chronic myelogenous leukaemia. Hematol Oncol.

[4] Light RW (2013). The Light criteria: the beginning and why they are useful 40 years later. Clin Chest Med.

[5] Bergeron A, Réa D, Levy V (2007). Lung abnormalities after dasatinib treatment for chronic myeloid leukemia: a case series. Am J Respir Crit Care Med.

[6] Quintás-Cardama A, Kantarjian H, O'brien S, Borthakur G, Bruzzi J, Munden R, Cortes J (2007). Pleural effusion in patients with chronic myelogenous leukemia treated with dasatinib after imatinib failure. J Clin Oncol.

[7] Shahed FH, Mamun-Al-Mahtab Mamun-Al-Mahtab, Rahman S (2016). he evaluation of serum ascites albumin gradient and portal hypertensive changes in cirrhotic patients with ascites. Euroasian J Hepatogastroenterol.

[8] Li A, Poon L, Khoo KL, Seet JE, Sinha AK, Lee P (2015). A man with pleural effusion and ascites. Chest.

[9] Zhang J, Tingle L, Nair R (2009). Discovering the elusive underlying cause of a bilateral effusion combined with ascites. Proc Bayl Univ Med Cent.

[10] Hou W, Sanyal AJ (2009). Ascites: diagnosis and management. Med Clin North Am.

[11] Inan I, De Sousa S, Myers PO, Bouclier B, Dietrich PY, Hagen ME, Morel P (2008). Management of malignant pleural effusion and ascites by a triple access multi perforated large diameter catheter port system. World J Surg Oncol.

[12] Ottmann O, Saglio G, Apperley JF (2018). Long-term efficacy and safety of dasatinib in patients with chronic myeloid leukemia in accelerated phase who are resistant to or intolerant of imatinib. Blood Cancer J.

[13] Brown JT, Beldorth IJ, Laosinchai-Wolf W (2019). Analytical validation of a highly sensitive, multiplexed chronic myeloid leukemia monitoring system targeting BCR-ABL1 RNA. J Mol Diagn.

[14] Ferreiro L, San-José E, Suárez-Antelo J, Valdés L (2016). Dasatinib-induced pleural effusion: chylothorax, an option to consider. Ann Thorac Med.

[15] Krauth MT, Herndlhofer S, Schmook MT, Mitterbauer-Hohendanner G, Schlögl E, Valent P (2011). Extensive pleural and pericardial effusion in chronic myeloid leukemia during treatment with dasatinib at 100 mg or 50 mg daily. Haematologica.

[16] Mishra AK, Sahu KK, Kaul S, Lal A (2020). Dasatinib induced pleuro-pericardial effusion. Acta Biomed.

[17] Hayes PC, Davis JM, Lewis JA (1990). Meta-analysis of value of propranolol in prevention of variceal haemorrhage. Lancet.

